# Editorial: Insights in: organizational psychology

**DOI:** 10.3389/fpsyg.2023.1304840

**Published:** 2023-10-27

**Authors:** Darren C. Treadway, Gabriele Giorgi, Monica Thiel

**Affiliations:** ^1^College of Hospitality and Tourism Management, Niagara University, Lewiston, ME, United States; ^2^Faculty of Psychology, European University of Rome, Rome, Italy; ^3^Asian Institute of Management, Makati, Philippines

**Keywords:** leadership, innovation, collective intelligence, job performance, job satisfaction

Since organizational psychology is a broad and evolving discipline, the topic editors are pleased to announce sixteen articles that highlight new insights into how leadership, collective intelligence, intellectual capital, innovation, job performance, satisfaction advance the tradition, nature, and research methods of organizational psychology. The articles showcase a global perspective of new insights in organizational psychology from Asia, Europe, Middle East, Africa, North America, and Oceania. The authors explored new insights in organizational psychology through conceptual analysis, qualitative and empirical research, a brief research report, and a systematic review.

There is a continual focus to expand and provide new insights on leadership theory and research (Lord et al., 2017). The authors focused on leadership directly and indirectly to provide new insights on evolving psychological mechanisms and psychological processes (van Vugt and Ronay, 2014) in organizational psychology. The editorial is organized through 4 topics namely, leadership, collective intelligence and intellectual capital, innovation, and job performance and satisfaction.

## Leadership

van Niekerk highlights the importance of psychosocial factors and elevated stress levels that limit a flourishing multi-cultural environment, stakeholder engagement and leader-follower relationships. The author recommends that organizations should promote a multi-cultural team to counteract elevated stress and to better manage psychosocial factors in an organization. Zhao et al. extend the literature on authoritarian leadership by providing a new perspective for authoritarian leadership practice. In comparison to previous research studies, the authors findings indicate authoritarian leadership generates positive employee wellbeing and creativity. Haar and de Jong explored the dark side of leadership personality to provide new insights on how the dark side of leadership could benefit an organization's performance rather than decreasing organizational performance. Latent transition analysis is introduced by Zyberaj et al. for helping organizational psychology researchers to analyze longitudinal data through an applied example utilizing psychological capital and leader-member exchange. The author findings indicates psychological capital is more likely to occur when leader-member exchange is high rather than low. Therefore, high leader-member exchange works hand-in-hand with high psychological capital.

## Collective intelligence and intellectual capital

Janssens et al. extends the collective intelligence literature and research through a conceptual analysis on the dynamic granular tensions between the needs of the environment with the collective team behaviors over time. The authors explored various methods to help organizational psychology researchers unpack micro-level team behavior. Senawi and Osmadi research study findings reveal that relational capital plays a significant role with intellectual capital for improving property tax reassessment activities. Overall, the attitudes of local government officials must align with relational capital and intellectual capital for successful property tax reassessment performance.

## Innovation

From a new Chinese perspective, Fan et al. research study findings extend self-determination theory to reveal employees' perceptions on organizational support has positive and profound effects on employees' proactive innovative behavior through the satisfaction of basic psychological needs such as autonomy, competence, and relatedness. Liu and Zhang focuses on employees' paradox mindset on innovative performance through role breadth self-efficacy. Employees with a paradox mindset intentionally make innovative things happen through their own actions. Moreover, role breadth self-efficacy and individual ambidexterity play an important role in understanding how employees manage a paradox mindset and innovative performance. Song et al. provide new insights on how employees should manage innovation performance under time pressure. The authors research study findings indicate time pressure significantly improves innovation performance. Therefore, employees operating under time pressures should receive significant leadership support for improving innovative performance.

## Job performance and job satisfaction

Xu et al. investigated the interdependence of psychological capital, social capital and human capital, including the three capital's impact on job performance. The authors study findings discovered new insights on configuration and casual asymmetry from psychological capital, social capital and human capital that affect job performance. Consequently, Xu et al. challenged previous studies' symmetrical regression relationship findings and extended the relationship among psychological capital, social capital and human capital within intelligent career theory. Moreover, high psychological capital plays a key role in high job performance. Sanclemente et al. explored inconsistencies from previous research studies that predict workers' health levels in linear models. The authors research study focused on differences among service sub sectors through linear and non-linear relationships within task complexity, job autonomy, user contacts, time pressure, and psychological and physical symptoms of employees. Overall, Sanclemente et al. research study findings on non-linear relationships indicate medium levels of task complexity from job demands should not exceed greatly to mitigate increased negative impacts to foster service sector employees' physical and psychological well-being in job satisfaction and performance.

Levitats et al. provides new insights on unexplored contexts of emotional intelligence in the literature. Specifically, the authors explore the role played by emotionally intelligence in an organization's culture combined with supervisors' emotionally intelligent behaviors. The two-study research findings reveal process links between emotionally intelligent values and practices, and job demands between supervisor emotional intelligence behaviors that affect employee exhaustion and engagement. Chen Y. et al. systematic review provides deeper understanding on the relationship between pay for performance and job performance by highlighting the research studies that examine pay for performance and job performance in real work settings. The results of the systematic review complements the positive effects of pay for performance and job performance in work settings through contextual performance and task performance. In addition, the authors introduce two mediating variables namely, intrinsic motivation and pressure that integrates the positive and negative effects of pay for performance and job performance into one framework.

The aim of López-Cabrera et al. research study is to explore potential factors that promote job satisfaction between volunteers and regular paid staff in non-profit organizations. The research study contributes to new understandings of the mechanism that promotes greater satisfaction from volunteer workers vs. regular paid staff through role ambiguity, role conflict and job performance. Chen C. et al. research study provides new insights on positive outcomes of customer incivility that could trigger employees' customer service behavior to challenge and extend the literature stream's focus on negative outcomes and customer incivility. The authors provide a more comprehensive understanding how customer incivility influences employees' behavior and the implications for revenge behavior and customer service behavior and performance in the work environment. Uzum et al. combine crab barrel syndrome and social comparison theory as a new approach for identifying precursors of crab barrel syndrome. The authors' research study findings indicate through social comparison theory that type A personality precedes crab barrel syndrome, especially when the work environment is highly competitive. Consequently, job performance for type A personalities requires strengthening the employee's self-esteem with group support to decrease crab barrel syndrome.

In conclusion, the articles in this Research Topic provide examples of new insights that we find relevant and thought provoking for progressing further research into organizational psychology scholarship from multiple perspectives.

## Author contributions

MT: Writing—original draft. DT: Writing—review and editing. GG: Writing—review and editing.
